# Supplementary material for: An integrative taxonomic approach reveals Octopus insularis as the dominant species in the Veracruz Reef System (southwestern Gulf of Mexico)
Source: PeerJ. 2018 Dec 6;6:e6015. doi: 10.7717/peerj.6015 (PMC6286802; doi:10.7717/peerj.6015)
Supplement: Supplemental Information 1 [file peerj-06-6015-s001.docx]

**Supplementary data:**

**Table S1: Raw morphological data for male (M) and female (F) specimens of the Veracruz Reef System common octopus.**

| Parameter | **P7AA** | **P9AA** | **P8AA** | **P13AA** | **P12AA** | **P16AA** | **P15AA** | **P14AA** | **P15AA bis** | **P50** | **P51** | **P52** | **P53** | **P54** | **P11AA** | **P10AA** | **P26IE** | **P31IE** |
| --- | --- | --- | --- | --- | --- | --- | --- | --- | --- | --- | --- | --- | --- | --- | --- | --- | --- | --- |
| **Sex** | M | M | M | M | M | M | M | M | M | M | M | M | M | M | F | F | F | F |
| **ML** | 164,00 | 159,00 | 116,60 | 108,50 | 104,60 | 102,10 | 102,60 | 101,30 | 114,90 | 156,00 | 189,00 | 154,00 | 142,00 | 111,50 | 141,00 | 157,00 | 140,00 | 113,20 |
| **GC** | 10 | 10 | 8 | 9 | 11 | 10 | 10 | 10 | 10 | 10 | 10 | 9 | 9 | 9 | 10 | 9 | 10 | 10 |
| **TW** | 1629,10 | 1117,10 | 684,20 | 441,80 | 571,80 | 319,80 | 335,10 | 450,00 | 1206,20 | 113,20 | 1811,30 | 1336,30 | 1377,60 | 509,50 | 1307,70 | 1326,60 | 826,10 | 595,50 |
| **TL** | 577 | 542 | 478 | 478 | 393 | 405 | 375 | 444 | - | 613 | 696 | 618 | 518 | 426 | 569 | 630 | 515 | 543 |
| **MW** | 96,60 | 72,90 | 63,30 | 53,20 | 64,30 | 53,30 | 55,50 | 56,60 | 85,30 | 93,60 | 110,00 | 91,40 | 96,10 | 70,00 | 88,00 | 87,80 | 77,60 | 90,80 |
| **HW** | 65,30 | 46,90 | 44,40 | 34,80 | 39,00 | 35,80 | 33,10 | 41,70 | 57,60 | 47,00 | 52,30 | 59,00 | 50,00 | 38,50 | 61,90 | 53,70 | 42,60 | 32,60 |
| **AL1R** | * | 330 | 323 | 243 | * | * | 276 | * | 374 | 387 | 421 | * | 401 | 350 | 416 | * | 313 | 280 |
| **AL1L** | * | 340 | 315 | 325 | * | 292 | * | 320 | 379 | 488 | * | 433 | * | * | * | 481 | 278 | * |
| **AL2R** | * | 456 | 381 | * | 242 | * | 273 | * | 418 | 527 | * | * | 418 | 363 | 474 | 536 | 389 | 416 |
| **AL2L** | * | 422 | 358 | 343 | 322 | 277 | 282 | 364 | 335 | * | 501 | * | 446 | 253 | 417 | 484 | 366 | 175 |
| **AL3R** | 405 | 365 | 304 | 299 | 321 | 259 | 257 | 294 | 338 | 404 | 446 | 438 | 346 | 314 | * | * | 281 | 445 |
| **AL3L** | * | * | * | 388 | * | * | 293 | 349 | 382 | * | * | * | * | 346 | * | 439 | 412 | * |
| **AL4R** | * | 408 | 366 | * | * | 308 | 284 | * | 413 | * | 590 | * | * | 348 | 406 | 512 | 441 | 465 |
| **AL4L** | 471 | 406 | 350 | 318 | 317 | 314 | 256 | * | 432 | * | * | 539 | * | * | * | 502 | 275 | 462 |
| **ASC1R** | * | 178 | 210 | 162 | * | * | 173 | * | 208 | 190 | 205 | * | 188 | 219 | 235 | - | 165 | 103 |
| **ASC1L** | * | 178 | 199 | 186 | * | 203 | - | 210 | 207 | 197 | * | 228 | * | * | * | 240 | 125 | * |
| **ASC2R** | * | 210 | 227 | * | 172 | * | 162 | * | 227 | 210 | * | * | 210 | 196 | 243 | 249 | 212 | 158 |
| **ASC2L** | * | 217 | 196 | 199 | 175 | 170 | 183 | 222 | 197 | * | 190 | * | 200 | 203 | 221 | 232 | 124 | * |
| **ASC3R** | 131 | 113 | 116 | 116 | 125 | 117 | 115 | 132 | 132 | 110 | 121 | 146 | 103 | 131 | * | * | 148 | 175 |
| **ASC3L** | * | * | * | 214 | * | * | 170 | 225 | 145 | * | * | * | * | 196 | * | 200 | 202 | * |
| **ASC4R** | * | 208 | 226 | * | * | 113 | 198 | * | 232 | * | 257 | * | * | 245 | 217 | 263 | 214 | 208 |
| **ASC4L** | 233 | 213 | 222 | 196 | 191 | 201 | 175 | * | 232 | * | * | 267 | * | * | * | 249 | 160 | 219 |
| **LL** | 4,95 | 3,90 | 4,23 | 3,95 | 4,48 | 2,62 | 3,20 | 3,25 | 5,38 | 3,80 | 5,80 | 4,05 | 5,70 | 4,05 | - | - | - | - |
| **CL** | 2,50 | 1,75 | 2,00 | 1,78 | 2,08 | 1,10 | 1,64 | 1,75 | 3,15 | 1,55 | 3,13 | 1,95 | 2,78 | 2,30 | - | - | - | - |
| **TOL** | 18,00 | 17,30 | 15,50 | 13,60 | 13,20 | 10,80 | 12,30 | 12,90 | - | - | 14,20 | 13,20 | - | 13,80 | - | - | - | - |
| **AW** | 25,50 | 27,90 | 20,10 | 15,90 | 18,60 | 15,10 | 15,60 | 16,20 | 26,60 | 20,00 | 26,40 | 23,40 | 28,10 | 17,70 | 25,30 | 21,10 | 19,60 | 16,80 |
| **WD_A** | 69,80 | 58,20 | 50,20 | 42,80 | 52,20 | 37,80 | 38,20 | 44,10 | 55,90 | 70,00 | 74,90 | 61,80 | 56,30 | 40,00 | 50,40 | 64,30 | 55,80 | 36,70 |
| **WB_B** | 92,05 | 73,00 | 64,55 | 63,60 | 61,80 | 52,25 | 52,45 | 56,10 | 72,15 | 84,15 | 96,40 | 74,05 | 72,70 | 59,00 | 83,25 | 86,35 | 71,00 | 55,75 |
| **WD_C** | 113,00 | 95,15 | 82,15 | 77,90 | 72,70 | 62,90 | 68,85 | 77,20 | 81,75 | 100,50 | 118,60 | 85,65 | 88,15 | 75,65 | 92,80 | 99,00 | 88,10 | 90,15 |
| **WD_D** | 115,10 | 93,50 | 75,25 | 76,05 | 69,40 | 62,40 | 73,00 | 75,35 | 94,00 | 102,55 | 127,95 | 88,10 | 97,15 | 64,15 | 86,15 | 98,35 | 94,90 | 91,10 |
| **WD_E** | 92,20 | 71,40 | 65,20 | 40,55 | 52,80 | 54,20 | 57,70 | 66,50 | 77,30 | 77,00 | 104,00 | 75,90 | 92,20 | 51,40 | 70,90 | 84,50 | 70,70 | 73,00 |
| **FL** | 55,50 | 42,60 | 39,80 | 32,80 | 38,70 | 33,00 | 30,80 | 36,40 | - | 47,70 | 54,80 | 41,30 | 45,50 | 34,00 | 49,10 | 50,10 | 36,40 | 41,90 |
| **ELD** | 5,90 | 7,00 | 6,40 | 5,20 | 7,70 | 6,40 | 5,50 | 6,90 | - | 8,90 | 7,00 | 9,50 | 10,10 | 7,80 | 8,50 | 7,60 | 5,40 | 8,80 |
| **SpL** | 52,49 | 44,615 | - | - | 40,461 | - | - | 33,846 | - | - | 57,166 | 49,538 | - | - | - | - | - | - |
| **SpW** | 0,749 | 0,692 | - | - | 0,621 | - | - | 0,566 | - | - | 0,88 | 0,923 | - | - | - | - | - | - |
| **PA** | 73,1 | 56,6 | 46,9 | 41,9 | 45,1 | 38,3 | 35,3 | 45,2 | 58 | 55,4 | 77,5 | 60,9 | 60 | 37,3 | 60,6 | 61,3 | 57,9 | 69,8 |
| **nSD** | 12,5 | 11,9 | 9,6 | 8,6 | 9,1 | 7,2 | 8 | 7,8 | 11 | 13 | 16,7 | 12,3 | 14,8 | 9,8 | 12,1 | 12,2 | 10,4 | 9,5 |
| **eSD** | 15,9 | 15,3 | 12,5 | 10,4 | 11,9 | 9,5 | 9,1 | * | 15,8 | 16,7 | 19,8 | 16 | 18,9 | 11,7 | - | - | - | - |

Acronyms: ML, mantle length; GC, gill count; TW, total weight; TL, total length; MW, mantle width; HW, head width; AL, arm length; ASC, arm sucker count; LL, ligula length; CL, calamus length; TOL, terminal organ length; AW, arm width; WD, web depth; FL, funnel length; ELD, eye lens diameter; SpL, spermatophore length; SpW, spermatophore width; PA, pallial aperture; nSD, normal sucker diameter; eSD, enlarged sucker diameter. * = missing or regenerating arms; - = data not available. Measurements are in mm and weights in g.
